# Toluene Adsorption by Mesoporous Silicas with Different Textural Properties: A Model Study for VOCs Retention and Water Remediation

**DOI:** 10.3390/ma13122690

**Published:** 2020-06-12

**Authors:** Chiara Vittoni, Giorgio Gatti, Ilaria Braschi, Enrico Buscaroli, Giovanni Golemme, Leonardo Marchese, Chiara Bisio

**Affiliations:** 1 Department of Sciences and Technological Innovation and Interdisciplinary Nano-SiSTeMI Centre, University of Eastern Piedmont A. Avogadro, Viale T. Michel 11, 15121 Alessandria, Italy; chiara.vittoni@uniupo.it (C.V.); giorgio.gatti@uniupo.it (G.G.); leonardo.marchese@uniupo.it (L.M.); 2 Department of Agricultural and Food Sciences, University of Bologna, Viale G. Fanin 44, 40127 Bologna, Italy; enrico.buscaroli2@unibo.it; 3 Department of Environmental Engineering, University of Calabria, Via P. Bucci 45A, 87036 Rende, Italy; giovanni.golemme@unical.it; 4 CNR-SCITEC Istituto di Scienze e Tecnologie Chimiche “Giulio Natta”, Via G. Venezian 21, 20133 Milano, Italy

**Keywords:** adsorption, fuel-based pollutants, toluene, mesoporous silica, FT-IR spectroscopy

## Abstract

In this work, different mesoporous silicas were studied as potential sorbents for toluene, selected as a model molecule of aromatic organic fuel-based pollutants. Three siliceous materials with different textural and surface properties (i.e., fumed silica and mesoporous Santa Barbara Amorphous (SBA)-15 and Mobil Composition of matter (MCM)-41 materials) were considered and the effect of their physico-chemical properties on the toluene adsorption process was studied. In particular, FT-IR spectroscopy was used to qualitatively study the interactions between the toluene molecule and the surface of silicas, while volumetric adsorption analysis allowed the quantitative determination of the toluene adsorption capacity. The combined use of these techniques revealed that textural properties of the sorbents, primarily porosity, are the driving forces that control the adsorption process. Considering that, under real conditions of usage, the sorbents are soaked in water, their hydrothermal stability was also investigated and toluene adsorption by both the gas and aqueous phase on hydrothermally pre-treated samples was studied. The presence of ordered porosity, together with the different pore size distribution and the amount of silanol groups, strongly affected the adsorption process. In toluene adsorption from water, SBA-15 performed better than MCM-41.

## 1. Introduction

The presence of organic pollutants such as hydrocarbons in groundwater can severely affect environmental and human health [1]. Among these, aromatic molecules BTEX (i.e., benzene, toluene, ethylbenzene, and xylenes) can be found as they are components of gasoline and other petroleum products. Moreover, the water solubility of aromatic fuel-based pollutants (e.g., 1.77 and 0.52 g L^−1^ at 20 °C for benzene and toluene, respectively) further favors their presence in water bodies [2,3]. In order to limit this type of pollution, in the last years, research efforts have focused on the development of different technologies aimed at decontaminating groundwater from hydrocarbons such as air sparging, flushing, use of permeable reactive barriers (PRBs), sand filtration, and pump and treat methods [2]. One of the most promising approaches to depollute groundwater is represented by PRBs in that surface manipulation and further work by operators are not required. In addition, PRBs exploit the natural groundwater flow and no energy costs are requested [4]. In PRBs, different types of sorbent materials can be used for water depollution [5]. Among these, high-silica zeolites have been widely studied due to their selectivity, chemical and hydrothermal stability, mechanical strength, and long lifetime [6,7,8,9,10]. However, the application of zeolites for the removal of hydrocarbons is hindered by their limited adsorption capacity and the exclusion from adsorption of large molecules, whose diffusion through zeolite micropores is impeded [11]. To overcome these limitations, the literature reports different types of mesoporous solids for pollutants adsorption. In particular, ordered mesoporous silicas were studied due to their remarkable properties such as high specific surface area, large pore size, and high amount of surface functional groups that can be exploited for their excellent selectivity toward specific pollutants [12,13]. Mobil Composition of matter (MCM)-41, a mesoporous silica with high surface area (higher than 1000 m^2^/g) and large pore volume with highly ordered hexagonally packed cylindrical pores, was compared to selected hydrophobic zeolites such as silicalite-1 and zeolite Y silica as sorbents of some volatile organic compounds (VOCs) in the gas phase (i.e., benzene, carbon tetrachloride, and n-hexane) [14]. MCM-41 resulted in an interesting potential sorbent for VOCs at high concentrations due to the large accessible internal pore volumes, which are fillable at relative high pressures [14].

In addition, in the most recent literature, studies concerning the use of mesoporous ordered silicas for VOC adsorption were published, mainly focusing on silica surface functionalization with organic groups to improve VOC adsorption capacity [15,16]. For example, Zhou et al. studied amino-functionalized spherical mesoporous silicas as potential toluene adsorbent materials. They found that the modifications of the surface occurring by the amino-functionalization affects both the pore structure and surface properties of siliceous materials, improving their toluene adsorption capacity [17].

In this work, different siliceous materials with variable porosity were studied for potential application in VOC removal and groundwater depollution. In particular, silicas with different textural properties were tested as sorbents for the removal of toluene, chosen as a model molecule of aromatic hydrocarbons, from both the gas and water phase. In particular, commercial fumed silica (characterized by heterogeneous porosity) and long-range ordered materials such MCM-41 and Santa Barbara Amorphous (SBA)-15 (i.e., a mesoporous ordered silica with a hexagonal array characterized by larger mesopores and higher wall thickness compared to MCM-41) were considered sorbent materials.

It is known that MCM-41 silica exhibits poor hydrothermal stability in water at high temperatures due to collapse of the porous structure [18]. Considering that the materials are soaked in water during their usage as PRBs and, in most cases, once exhausted, are regenerated to be used again, the hydrothermal stability is as an important feature that promising adsorbents must have [5]. For these reasons, in this work, particular attention was devoted to the study of the hydrothermal stability of mesoporous silicas. After a detailed physico-chemical characterization aimed to understand the main structural, morphological, textural, and surface properties before and after the hydrothermal treatment, the study of toluene adsorption was carried out.

Different techniques were used to study the adsorption process from both qualitative and quantitative points of view in order to gain knowledge on the interactions between the adsorbent surface and aromatic compounds exploitable to improve VOC adsorption and wastewater remediation technologies. More in detail, the capacity of toluene adsorption of these solids, together with the type and strength of the host–guest interactions between toluene and the silica surface, was studied by a combination of FT-IR spectroscopy and volumetric analysis. The effect of the textural properties of the sorbents, mainly porosity, on toluene adsorption was also investigated in depth. Finally, the study of the toluene adsorption was also performed on materials after the hydrothermal treatment, in order to determine its effect (i.e., pore architecture modifications) on toluene adsorption properties. The investigation was carried out both in the gaseous and aqueous phase, in order to cover any possible application of these materials such as VOC adsorption and water remediation.

## 2. Materials and Methods

### 2.1. Materials

MCM-41 silica (CAS Number 7631-86-9) and fumed silica (Aerosil 380, CAS Number 112945-52-5) were purchased from Sigma Aldrich (St. Louis, MS, USA) and Evonik Industries (Essen, Germany), respectively.

SBA-15 silica was prepared following the synthesis method reported by Zhao [19]. In detail, 4 g of Pluronic P123 ((EO)_20_(PO)_70_(EO)_20_, MW = 5800, Sigma Aldrich, CAS Number 9003-11-6) was dissolved in 30 mL of deionized water for 24 h at 35 °C. Then, 120 mL of 2 M HCl aqueous solution (Sigma Aldrich) was added and the sol was stirred for 1 h at the same temperature. Then, 8.5 g of tetraethyl orthosilicate (TEOS, 99 wt.%, Sigma-Aldrich) was added and the obtained gel was placed under static conditions in a Teflon-lined autoclave at 60 °C for 24 h. The resulting white precipitate was filtered, washed with ultrapure water, and dried for 24 h. Finally, the powder was calcined at 550 °C for 5 h (heating rate of 1 °C/min). 

Toluene was purchased from Sigma-Aldrich with a purity of 99.9%.

### 2.2. Characterization Techniques

X-ray diffraction (XRD) patterns were obtained using an ARL XTRA48 diffractometer (Waltham, MA, USA) with Cu Kα radiation (λ = 1.54062 Å) with a 2θ range between 0.7 and 4° for SBA-15 and between 1.5 and 7° for MCM-41 with a scanning rate of 1° 2θ/min for both samples.

TEM images were collected on a JEOL 3010 high-resolution transmission electron microscope (Tokyo, Japan) operating at 300 kV. Before analysis, samples were dispersed by sonication in isopropanol and then deposited on carbon-coated grids.

Thermogravimetric measurements were recorded with a SETSYS Evolution TGA-DTA/DSC thermobalance (Caluire-et-Cuire, France). Samples were heated from room temperature (RT) to 1100 °C at a heating rate of 2 °C/min under oxygen flow at 100 mL/min.

The specific surface area (SSA) of silica samples was measured by means of nitrogen adsorption at 77 K in the pressure range between 1.04 × 10^−4^ and 1009 mbar using an Autosorb-iQ instrument (Quantachrome Instruments, Boynton Beach, FL, USA). Prior to adsorption, all samples were outgassed (final pressure 7 × 10^−4^ mbar) and thermally treated as follows: 1 h at 80 °C, 2 h at 120 °C, 2 h at 150 °C, and finally 10 h at 220 °C. The SSA of the samples was determined by the Brunauer-Emmett-Teller (BET) equation in the range 0.1–0.25 p/p_0_ of relative pressure. The obtained R^2^ values were 0.999939, 0.999413, and 0.999885 for fumed, MCM-41, and SBA-15 silica, respectively. The pore size distribution was also calculated by applying the Non-Local Density Functional Theory (NLDFT) cylindrical pore kernel in the desorption branch isotherms.

Toluene adsorption isotherms by the gas phase were obtained at 35 °C by volumetric analysis of vapor sorption employing an Autosorb-iQ instrument (Quantachrome Instruments). Prior to adsorption, the samples were outgassed (final pressure 7 × 10^−4^ mbar) and thermally treated as follows: 30 min at 50 °C, 30 min at 80 °C, 2 h at 120 °C, 2 h at 150 °C and, finally, 12 h at 220 °C, in order to remove completely adsorbed water.

Infrared spectra were collected on a Thermo Electron Corporation FT Nicolet 5700 spectrometer (Waltham, MA, USA) with 4 cm^−1^ resolution. Then, 32 scans for each spectrum acquisition were recorded. Self-supporting pellets of silica-fumed, MCM-41, and SBA-15 samples were obtained with a mechanical press at ca. 7 tons cm^−2^ and placed into an IR cell equipped with KBr windows permanently attached to a vacuum line (residual pressure ≤ 1 × 10^−3^ mbar), allowing all treatments and toluene adsorption/desorption experiments to be carried out in situ. Spectra of toluene adsorbed on solids were collected at the beam temperature (ca. 35 °C) on samples previously dehydrated. For fumed silica and MCM-41, the dehydration was performed in vacuum at RT, while for SBA-15, it was necessary to heat the sample until 150 °C under vacuum.

### 2.3. Hydrothermal Treatments

To study the hydrothermal stability of MCM-41 and SBA-15 samples, the solids (100 mg) were dispersed in 10 mL of water in a glass vial with a screw cap (Sigma Aldrich, 20 mL capacity) and the dispersion was heated at 50 °C by using a laboratory magnetic stirrer with a heating plate hotplate for different times to accelerate the material degradation. The temperature was kept constant by inserting vials in a sand bath. Two aliquots of each sample were prepared and placed in water for 8 and 36 h, respectively. After that, samples were recovered by filtration and then dried at 60 °C for 24 h. Samples recovered after 8 h of hydrothermal treatment were named MCM-41_8h and SBA-15_8h, whereas those treated for 36 h were coded MCM-41_36h and SBA-15_36h, respectively. 

### 2.4. Toluene Adsorption from Aqueous Phase

#### 2.4.1. Adsorption Kinetics

One liter of toluene solution (100 mg L^−1^) was prepared in milliQ® water. Ten mg of MCM-41 or SBA-15, before and after 8 and 36 h of hydrothermal treatment, was placed in contact with 100 mL of the toluene solution in a 120 mL Erlenmeyer flask tightly closed with a silicon stopper to limit toluene evaporation and to allow withdrawals of the suspension by a syringe needle without opening the flask. The suspension was kept under magnetic stirring at RT. After 0.25, 0.5, 1, 2, 4, and 6 h, 0.75 mL aliquots of each suspension were withdrawn under stirring with a syringe and filtered through 0.20 µm hydrophilic PTFE syringe filters. The filtered solution was placed into high-performance-liquid-chromatography (HPLC) vials for quantitative analysis and diluted 1:1 with acetonitrile (CAS 75-05-8, LC grade, Sigma Aldrich) to limit toluene evaporation during the chromatographic analysis. Each kinetics was replicated three times. A control trial was performed in the absence of sorbent and the toluene concentration was evaluated in the aqueous solution after the same time intervals and filtration step.

#### 2.4.2. Adsorption Isotherms

Toluene solution was prepared in milliQ® water in the 20–80 mg L^−1^ range of concentration (namely 20, 40, 60, and 80 mg L^−1^). Ten mg of MCM-41 or SBA-15, before and after 8 and 36 h of hydrothermal treatment, was placed in contact with 100 mL of the toluene solutions at different concentrations in 120 mL Erlenmeyer flasks tightly closed with a silicon stopper to limit toluene evaporation, and the suspensions were kept under magnetic stirring at RT. After 4 h of contact, 1 mL of aliquots of each suspension was withdrawn by a syringe and processed as reported in the adsorption kinetics session. Each isotherm was replicated three times.

#### 2.4.3. HPLC Analysis

An HPLC system assembled with a Jasco 880-PU Intelligent pump, a Jasco AS-2055 plus Intelligent Sampler, a Jasco 875-UV Intelligent UV–vis Diodarray detector set at 206 nm (Alexandria, VA, USA), and Borwin v 1.2160 chromatography software were used to determine the toluene concentration in the solutions resulting from adsorption kinetics and isotherm trials. A C18 Luna 5 µm HILIC 150 mm analytical column (Phenomenex, Torrance, CA, USA) was kept in a column oven at 35 °C (Jones Chromatography model 7971) and eluted with a mixture of HPLC grade H_2_O and CH_3_CN (45 and 55% by volume, respectively) at 1 mL min^−1^ of flow rate. Under these chromatographic conditions, the retention time of toluene was 4.3 min. The toluene quantitative determination of toluene, measured as an average value of triplicate injections, was performed by using external standard (r_working curve_ = 0.99999).

## 3. Results and Discussion

### 3.1. Physico-Chemical Characterization

**Figure 1:**
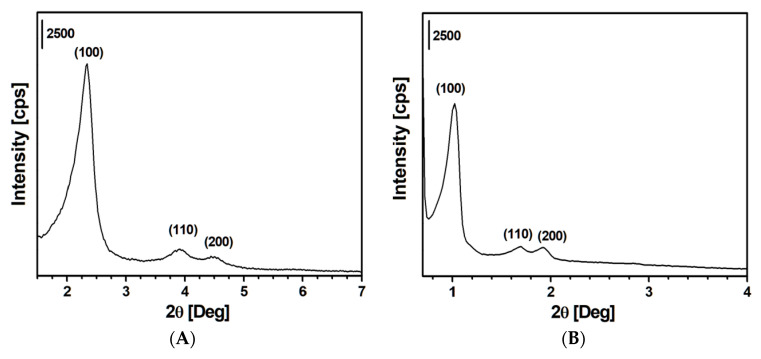
Powder X-ray diffraction patterns of Mobil Composition of matter (MCM)-41 (**A**) and Santa Barbara Amorphous (SBA)-15 (**B**) silicas.

To understand the structural properties of ordered mesoporous silicas, powder XRD analysis was performed. The XRD patterns of SBA-15 and MCM-41 silicas are reported in Figure 1.

The diffraction pattern of the MCM-41 sample (Figure 1A) presents three Bragg reflections at 2.34, 3.90, and 4.50° 2θ, corresponding to (100), (110), and (200) planes that are characteristic of ordered mesoporous silica. The SBA-15 sample (Figure 1B) shows a similar trend with reflections at 1.01, 1.70, and 1.92° 2θ, corresponding to (100), (110), and (200) planes, respectively. This pattern indicates that the adopted synthesis procedure formed the typical 2-d hexagonal (p6mm) mesoporous SBA-15 silica phase [19,20,21]. The ordered porosity with hexagonal symmetry of both sample is also appreciable in TEM micrographs (Figure S1 in Supporting Information). On the contrary, fumed silica did not present any reflections (data not shown for the sake of brevity), as it is not characterized by an ordered array of pores.

**Figure 2:**
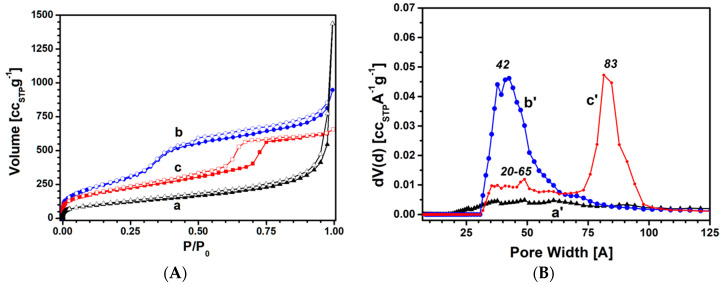
(**A**) N_2_ adsorption (full symbols) and desorption (empty symbols) isotherms at 77 K of fumed, MCM-41, and SBA-15 silica (curves a, b, and c, respectively); (**B**) pore size distribution of fumed, MCM-41, and SBA-15 silica (curves a’, b’, and c’, respectively).

N_2_ adsorption-desorption isotherms at 77 K were also performed to gain information about textural properties of the sorbents. SSA and pore size distribution of the silica samples are shown in Figure 2. Textural features of the samples are reported in Table 1.

The fumed silica isotherm (Figure 2A, curve a) is of type II and shows an H3-type hysteresis loop [22,23]. The narrow hysteresis loop suggests the presence of two kinds of aggregation related to the presence of primary particles, produced by synthesis, and secondary particles, formed by aggregation and/or agglomeration of primary particles [22].

The N_2_ adsorption isotherm of the MCM-41 sample (Figure 2A, curve b) is of type IV (b), characteristic of hexagonal ordered mesoporous silicas with both conical and cylindrical mesopores with small pore width [23,24]. The narrow hysteresis of type H4, observable in a 0.5–0.9 p/p_0_ range, confirmed the presence of a fraction of cylindrical mesopores [25,26,27]. From Figure 2B, it can be observed that MCM-41 has a wide pore size distribution (curve a’) between 30 and 80 Å, with a maximum centered at ca. 42 Å and an associated mesopore volume of 0.90 cm^3^ g^−1^.

The SBA-15 sample (Figure 2A, curve c) presents an isotherm of type IV(a), typical of mesoporous silicas with ordered hexagonal arrays [23]. The SBA-15 hysteresis loop presents a two-step capillary condensation in the range of 0.5–0.8 p/p_0_, classified as type H1 and H2, which suggests the co-presence of cylindrical and cage-like pores [28,29]. Indeed, the sample presents two different pore families (see Figure 2B, curve c’): A heterogeneous pore family from 20 to 65 Å and a homogeneous family between 65 and 100 Å with a maximum centered at 83 Å.

The main textural properties of the three silicas obtained by N_2_ physisorption analysis are reported in Table 1.

As reported in Table 1, fumed silica is characterized by an SSA of 412 m^2^ g^−1^ with a total pore volume of 1.47 cm^3^ g^−1^. The volume for the mesopore fraction of 0.31 cm^3^ g^−1^ is related to the heterogeneous pore size distribution from 20 to 100 Å. MCM-41 has an SSA of 1103 m^2^ g^−1^ and total pore volume of 1.31 cm^3^ g^−1^. The volume of the mesopore fraction between 20 and 100 Å is 0.98 cm^3^ g^−1^. The SBA-15 sample presents an SSA of 761 m^2^ g^−1^ and a total pore volume of 0.96 cm^3^ g^−1^ associated with the two different families of pores in the 20–65 and 65–100 Å range, with related mesopore fractions of 0.31 and 0.55 cm^3^ g^−1^, respectively. The SSA of both MCM-41 and SBA-15 is higher than that of fumed silica.

Surface properties of the silica samples were studied by FT-IR spectroscopy. The spectra of fumed, MCM-41, and SBA-15 silica samples (Figure S2 curves a, b, and c, respectively, in the Supporting Information) are characterized by a sharp peak at ca. 3745 cm^−1^ due to isolated silanols, and a broad absorption in the 3720–3200 cm^−1^ region, with a maximum at around 3530 cm^−1^, due to hydrogen-bonded silanols [30,31]. As the amount of silanols is a key parameter that can strongly influence the adsorption of toluene [8], quantification of the silanols in the samples was performed by using thermogravimetric analysis (Figure S3 and Equation (S1) in Supporting Information). From this study, it was derived that the total amount of silanols is 2.5, 2.0, and 2.8 OH/nm^2^ for fumed, MCM-41, and SBA-15 silica, respectively.

### 3.2. Monitoring the Hydrothermal Stability of SBA-15 and MCM-41 Silicas

The stability of the mesoporous ordered samples in the presence of water and the possible modifications occurring after different contact times with water were studied by using different experimental techniques. To speed up the process, the sorbent powders were soaked in warm water (50 °C) for different time periods (i.e., 8 and 36 h) [32].

XRD analysis on samples before and after hydrothermal treatment is reported as Supporting Information. The diffraction pattern of SBA-15 samples (Figure S4B) treated for 8 and 36 h is not significantly modified with respect to that of the untreated sample, thus suggesting that the hexagonal ordered array of pores is preserved after treatment.

By contrast, the diffraction pattern of the MCM-41 sample (Figure S4A in Supporting Information) is strongly altered by the hydrothermal treatment. In fact, it is possible to notice that the reflection associated with the (100) plane is widened and decreased in intensity, while (110) and (200) reflections are completely disappeared after 8 and 36 h of treatment (Figure S4A, curves a’ and a’’). This suggests that MCM-41 partially lost the hexagonal pore array after the treatment. It is already reported in the literature that the MCM-41 structural properties can be drastically affected by exposure to water or even to air humidity and that its destructuration, and subsequent water dissolution, is relatively high compared to SBA-15 silica [33]. The low hydrothermal stability of MCM-41 silica is due to the lower wall thickness (0.5 nm) compared to that of SBA-15 (2.8 nm). Details of the determination of the wall thickness are reported as Supporting Information [33,34].

The effects of 36 h of hydrothermal treatment on MCM-41 were also investigated by TEM analysis (see Figure S5 in the Supporting Information). The analysis confirmed a significant decrease in the order of the hexagonal pore lattice, accompanied by the formation of irregular “holes,” thus indicating partial destructuration of the material.

**Figure 3:**
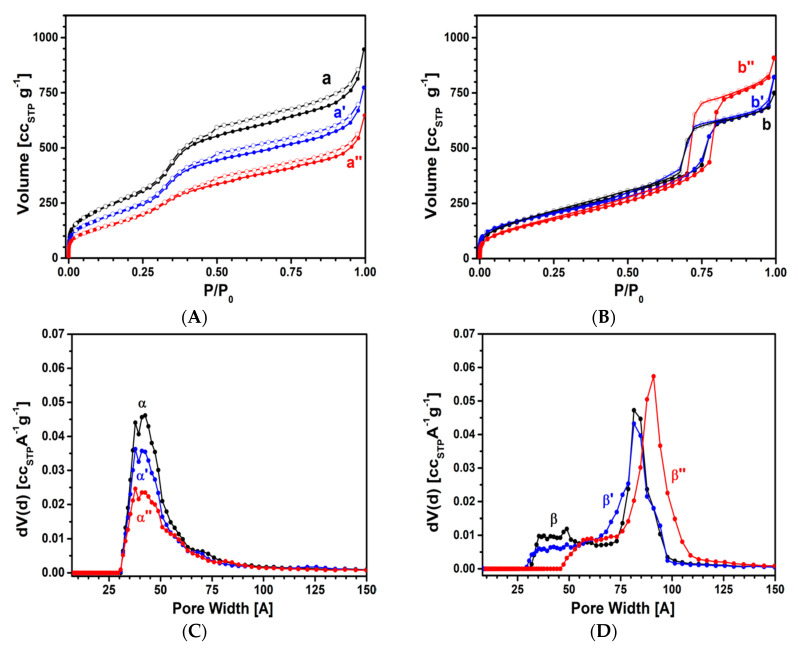
N_2_ adsorption (full symbols) and desorption (empty symbols) isotherms of MCM-41 (a), MCM-41_8h (a’), MCM-41_36h (a’’) (**A**), SBA-15 (b), SBA-15_8h (b’), SBA-15_36h (b’’) (**B**); pore size distributions of MCM-41 (α), MCM-41_8h (α’), MCM-41_36h (α’’) (**C**), SBA-15 (β), SBA-15_8h nh89 (β’), SBA-15_36h (β ’’) (**D**).

Modification of the textural properties of the ordered porous samples after the hydrothermal treatment was investigated by N_2_ physisorption analysis. Specific surface area (SSA) and pore size distribution are reported in Figure 3 and Table 1.

The N_2_ adsorption isotherms of MCM-41 and SBA-15 silicas are shown in Figure 3A,B, respectively. All samples, before and after hydrothermal treatment, exhibit type IV isotherms with capillary condensation steps occurring at a partial pressure between 0.5 and 0.9 p/p_0_ with narrow hysteresis of type H4 for MCM-41 samples (Figure 3A, curve a) and between 0.65 and 0.8 p/p_0_ with type H1 and H2 hysteresis for SBA-15 samples (Figure 3B, curve b) [25,26,27,28,29].

The overall textural properties of MCM-41 (Figure 3A,C) after the hydrothermal treatment are progressively modified with respect to the bare sample: After 8 h (Figure 3A, curve a’), the SSA decreases from 1103 to 888 m^2^ g^−1^ with a decrease in the pore volume passing from 1.31 to 1.07 cm^3^ g^−1^. After a prolonged treatment (36 h, Figure 3B curve a’’), a further decrease in the SSA to 607 m^2^ g^−1^ and of the pore volume to 0.87 cm^3^ g^−1^ is observed.

In Figure 3C, it is possible to notice that the pore family from 20 to 100 Å, with a maximum centered at 42 Å (Figure 3C, curve α) and an associated pore volume of 0.98 cm^3^ g^−1^, is still observed after 36 h of treatment (Figure 3C, curve α’’). Nevertheless, the volume of this family of pores reduces progressively after treatments to 0.79 and 0.63 cm^3^ g^−1^ after 8 and 36 h, respectively (Figure 3C, curves α’ and α’’). This behavior could be associated with partial hydrolysis of MCM-41 silica walls. Nevertheless, it is worth noticing that the wall thickness of the MCM-41 is not altered during the treatment in water at 50 °C (see Table 1).

Concerning SBA-15, after the hydrothermal treatment, it is possible to notice that for the SBA-15_8 h sample (Figure 3B, curve b’), the isotherm is similar to that of the untreated material (Figure 3B, curve b). Compared to untreated material, the SSA slightly decreases from 761 to 689 m^2^ g^−1^, whereas the pore volume increases from 0.96 to 1.14 cm^3^ g^−1^. A more pronounced modification of the N_2_ isotherm is visible for the SBA-15_36h (Figure 3B, curve b’’ and Figure S6 in Supporting Information). For this sample, the SSA is reduced to 634 m^2^ g^−1^ while the pore volume is further increased to 1.30 cm^3^ g^−1^, as reported in Table 1. This modification occurs mainly through a reduction in the smallest mesopores (as it can also be observed from the isotherms reported in semi-logarithmic scale, see Figure S6 in Supporting Information).

To better understand this particular behavior, it is useful to focus our attention on the pore size distribution (Figure 3D). After 8 h of hydrothermal treatment (Figure 3D, curve β’), the pore volume of the mesopore family between 20 and 65 Å decreases from 0.31 to 0.24 cm^3^ g^−1^. This pore volume further decreases after 36 h of treatment (Figure 3D, curve β’’) to 0.14 cm^3^ g^–1^. As already explained, SBA-15 presents a family of pores in the 65–100 Å range with a maximum at ca. 83 Å. Interestingly, the volume (0.88 cm^3^ g^−1^) and the size of this pore family increase (the maximum is now centered at ca. 91 Å), thus indicating the expansion of the pore size due to the hydrothermal treatment. This feature is also reported by Celer and co-workers [35]. In this study, after hydrothermal treatment of the SBA-15 sample at 100 °C for different times (6 h–8 days), an increase in the pore volume and pore size with a consequent decrease in SSA, due to the disappearance of the small mesopores between 20 and 65 Å and the growth of bigger mesopores, was observed. These evolutions would be due to deconstruction of the pore silica wall by hydrolysis, followed by recondensation of hydrolyzed silica to form bigger pores [35]. In our study, the enlargement of SBA-15 pore diameter after 36 h of hydrothermal treatment (Figure 3D) is accompanied by a clear shortening of the unit cell parameter a_0_ (Figure S4 in Supporting Information), with a consequent reduction in wall thickness, passing from 2.8 to 1.0 nm, after 36 h of treatment (Table 1).

The modification of the surface of silicas because of the hydrothermal treatment was also followed by FT-IR spectroscopy and thermogravimetric analysis (see Figures S7 and S8, and Table S1 as Supporting Information). From these analyses, it is observed that the amount of –OH groups per nm^2^ after 8 h of hydrothermal treatment increases for both mesoporous materials due to the progressive hydrolysis reactions on the silica surface. This effect is more evident for MCM-41 after 8 h of hydrothermal treatment compared to the SBA-15 sample, likely due to the thinner pore walls of MCM-41.

### 3.3. Monitoring the Interactions of Toluene on Fumed, MCM-41, and SBA-15 Silicas

The role of surface groups on the adsorption properties of the silica samples with different textural properties was studied by FT-IR and volumetric measurements of adsorbed toluene from the gas phase.

#### 3.3.1. Toluene Adsorption on Pristine Samples

**Figure 4:**
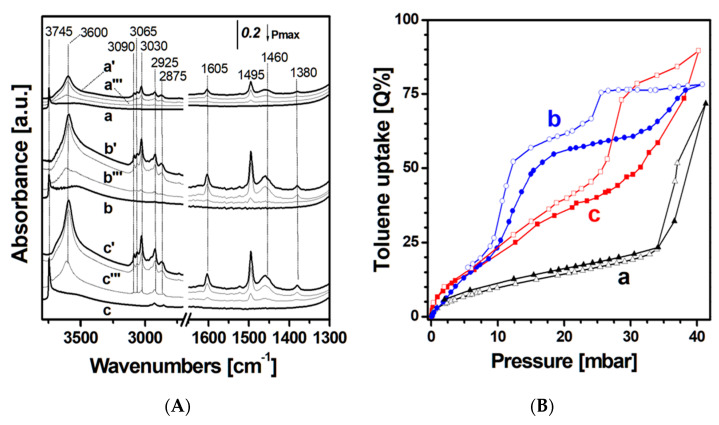
(**A**) FT-IR spectra of fumed, MCM-41, and SBA-15 silica samples: Spectra outgassed at RT for 1 h (curves a, b, and c respectively); spectra collected after dosage of toluene at 30 mbar on the same supports (curves a’–c’); 15 mbar (curves a’’–c’’) and 1 mbar (curves a’’’–c’’’). (**B**) Toluene volumetric adsorption (full symbols) and desorption (empty symbols) isotherms of toluene adsorption at 35 °C on fumed (a, triangles), MCM-41 (b, circles), and SBA-15 silica (c, squares). Prior to analysis, silica samples were outgassed under vacuum at 220 °C for 12 h.

The comparison of selected IR spectra related to the adsorption of toluene on fumed, MCM-41, and SBA-15 silicas is reported in Figure 4A. For the sake of clarity, a detailed description of the FT-IR spectra obtained after the adsorption of 30 mbar of toluene and subsequent gradual decrease in toluene pressure on the silicas is reported in Figure S9 and Table S2 as Supporting Information.

As a general feature, the admission of toluene on silica samples (Figure 4A, curves a’–c’) results in a progressive disappearance of the band related to isolated silanol species at 3745 cm^−1^ and into the formation of an intense band centered at 3600 cm^−1^, which is associated with π interactions between silanol species and toluene molecules (associated Δυ_OH_: ca. 145 cm^−1^) [8,36].

Moreover, upon toluene admission, all the bands related to the molecular vibrations (i.e., of both the aromatic ring and the methyl group) are also observed. More in detail, bands at 3090, 3065, and 3030 cm^−1^, related to C–H stretching modes of the toluene aromatic ring, and signals at 2925 and 2875 cm^−1^, assigned to C–H stretching modes of the toluene methyl group, are visible. In addition, a sharp signal at 1605 cm^−1^, due to the quadrant stretching mode of the monosubstituted ring C=C bond, a band at 1495 cm^−1^, associated with the semicircular stretching vibration of the monosubstituted aromatic ring, and, finally, bands at 1460 and 1380 cm^−1^, corresponding to the out-of-phase and in-phase deformations of the methyl group are also observable [8].

By analyzing the intensity of the IR bands relative to toluene adsorbed at high pressure (particularly bands at 1605 and 1380 cm^−1^), it is possible to obtain a semi-quantitative indication of the amount of toluene adsorbed by the siliceous materials. At 30 mbar, it can be clearly noticed that the ordered mesoporous samples retain a higher amount of toluene compared to fumed silica (Figure 4A, curve a’), whereas it seems that MCM-41 and SBA-15 silicas (Figure 4A, curves b’ and c’) adsorb a comparable amount of toluene. For all samples, by diminishing the toluene pressure to 15 (Figure 3A, curves a’’–c’’) and 1 mbar (Figure 3A, curves a’’’–c’’’), a progressive decrease in the IR bands of toluene and a progressive restoring of the band due to isolated SiOH species are visible. This is especially evident at low toluene pressure, thus indicating that SiOH species have a crucial role in adsorbing toluene at low pressure, whereas at high pressure, host-guest and guest-guest van der Waals interactions are driving the process, as already observed for high-silica zeolites [8].

In order to gain more detailed information on the amount of toluene adsorbed by the siliceous materials, volumetric isotherms at 35 °C of toluene vapor were performed (Figure 4B). Different regimes can be observed by the analysis of the volumetric isotherms for the three silicas.

The volumetric isotherm of fumed silica (Figure 4B, a) presents a narrow hysteresis loop at high toluene pressure related to condensation in porosities given by the aggregation of the particles. Three regimes can be observed in the isotherm: The isotherm is indeed steep until 2 mbar, and then the slope decreases up to ca. 35 mbar, when the slope starts to increase again. The curve does not reach a plateau, thus suggesting that the pore saturation does not occur. The uptake at 2 mbar is ca. 5 Q%, and at 35 mbar, it is 24 Q%, where:

(1) $$Q\%=\frac{m\;\mathrm{adsorbed}\;\mathrm{toluene}\;\left(\mathrm{mg}\right)}{100\;\mathrm{mg}\;\mathrm{of}\;\mathrm{sample}}$$

The third regime at higher pressures (>35 mbar) presents an uptake of 71 Q%. This behavior could be associated with the filling of heterogeneous porosity between 20 and 300 Å given by the aggregation of particles (see pore size distribution in Figure 2B, curve a’).

The volumetric isotherm of toluene adsorbed on MCM-41 (Figure 4B, curve b) presents three regimes of adsorption and two hysteresis loops formed in the range of 9–25 and 25–40 mbar due to a capillary condensation of the toluene molecules inside the pores. The curve appears rapid until 9 mbar, and then the slope increases up to ca. 15 mbar, when the adsorption curve gradually tends to a first plateau at ca. 25 mbar. By analyzing the IR data (data not shown for the sake of brevity), the rapid increase in toluene uptake until 9 mbar, corresponding to ca. 20 Q%, can mainly be associated with the interaction of surface silanols with toluene. The first plateau at 25 mbar, with an uptake of ca. 59 Q%, is associated with the filling of the fraction of mesopores with a diameter lower than 40 Å (see pore size distribution in Figure 2B, curve b’). At pressures higher than 25 mbar, the slope increases again and progressively until 45 mbar, where the overall toluene uptake is ca. 78 Q%. This last adsorption step is probably associated with the filling of the fraction of mesopores with dimensions between 40 and 80 Å.

The volumetric isotherm of toluene adsorbed on SBA-15 (Figure 4B, curve c) presents three regimes of adsorption and two hysteresis loops formed in the range between 9–25 and 25–40 mbar. The isotherm is steep until 1 mbar of toluene with a corresponding uptake of 7 Q%. The uptake of SBA-15 at 1 mbar of toluene is higher (+4%) with respect to that of the MCM-41 sample. This could be associated with the higher amount of isolated silanol species: 2.8 and 2.0 OH/nm^2^ for SBA-15 and MCM-41 sample, respectively. The dependence of toluene uptake to silanols is reported in Figure S10 as Supporting Information. At pressures higher than 1 mbar, the slope decreases up to ca. 27 mbar where the uptake is ca. 43 Q%. This second adsorption step is probably associated with the filling of the heterogeneous family pores from 20 to 65 Å (see pore size distribution in Figure 2B, curve c’). At pressures higher than 27 mbar, the slope increases up to ca. 31 mbar, and then increases again progressively until 40 mbar, where the overall toluene uptake is ca. 90 Q%. This third adsorption step is likely associated with the filling of the homogeneous pore family between 65 and 100 Å (Figure 2B, curve c’).

From the consideration reported above, it appears clear that textural properties, especially porosity, play an important role in determining the adsorption properties of used supports. In general, the presence of ordered porosity seems to favor the toluene adsorption, as SBA-15 and MCM-41 show a higher uptake in all the pressure ranges compared to fumed silica. Furthermore, the different pore size distribution characterizing SBA-15 and MCM-41 silicas deeply influences toluene adsorption. At low pressures (ca. 27 mbar), MCM-41 showed a higher adsorption capacity compared to SBA-15 due to the filling of the mesopores between 20 and 65 Å, whose correlated pore volume resulted higher compared to SBA-15 (see Table 2). Instead, at higher pressures (ca. 45 mbar), SBA-15 showed a higher adsorption capacity compared to MCM-41 due to the filling of the pores between 65 and 100 Å, with a correlated pore volume higher compared to MCM-41 (see Table 2).

#### 3.3.2. Toluene Adsorption on Silica Samples Treated Under Hydrothermal Conditions

FT-IR adsorption experiments were also carried out on the mesoporous ordered silica samples (SBA-15 and MCM-41) after hydrothermal treatments. 

**Figure 5:**
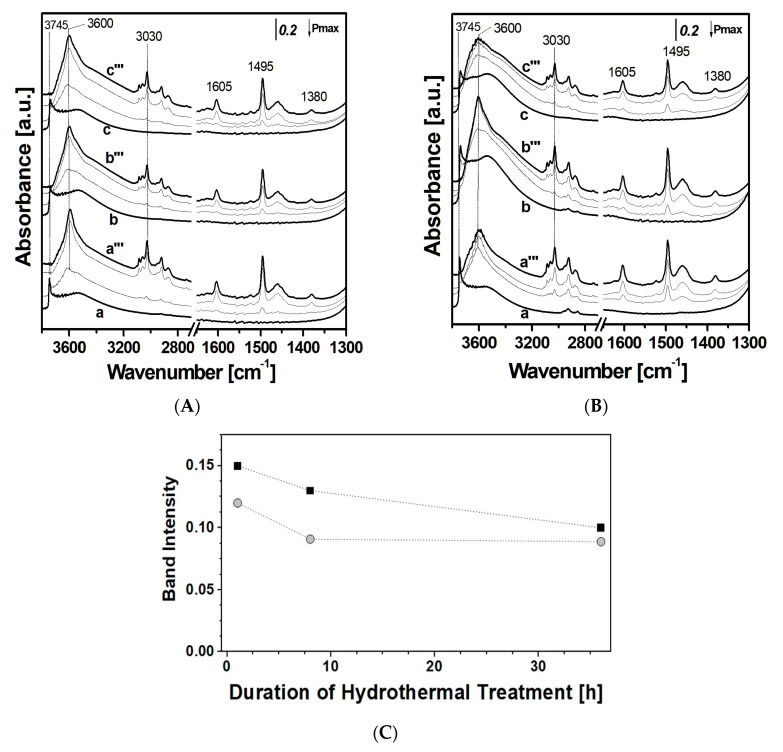
FT-IR spectra of toluene adsorbed on MCM-41, MCM41_8h, and MCM41_36h (**A**) and on SBA-15, SBA-15_8h, and SBA-15_36h (**B**). For each sample, the spectra of the silica outgassed at room temperature (RT) for 1 h is reported (curves a, b, and c for bare sample, 8 and 36 h of treatment, respectively); spectra collected after dosage of toluene at pressure of 1 mbar on the same supports (curves a’–c’); 5 mbar (curves a’’–c”) and 15 mbar (curves a’’’–c’’’) are also reported. Intensity of the band at 1605 cm^−1^ of toluene at pressure of 1 mbar on MCM-41 (grey circles) and SBA-15 (black squares) samples, before and after the hydrothermal treatment (**C**).

In Figure 5, selected FT-IR spectra of toluene adsorbed on MCM-41 (Frame A) and SBA-15 silica samples (Frame B) before and after hydrothermal treatments are reported. For a better comprehension, the intensity of the band at 1605 cm^−1^ due to the stretching mode of the mono-substituted ring C=C bond of toluene is reported in Frame C for all samples.

As a general comment, the spectra of toluene adsorbed on treated MCM-41 (Frame A) and SBA-15 (Frame B) materials show features already described in the case of original materials. These absorbance data were also used to derive information about the toluene uptake, as the intensity of the bands at 1605 cm^−1^, relative to toluene molecule (Frame C), is proportional to the amount of toluene adsorbed [10].

From Figure 5C, it is possible to notice that the SBA-15 sample shows a progressive slight decrease in the amount of toluene adsorbed after 8 and 36 h of treatment compared to the untreated sample. This effect could be due to the decrease in the surface area (689 and 634 m^2^ g^−1^ respectively for SBA-15_8h and SBA-15_36h) compared to the untreated sample (728 m^2^ g^−1^). For MCM-41 silica instead, the decrease in toluene adsorption is more pronounced, probably due to the heavy modifications of textural properties after the hydrothermal treatment (i.e., decrease in the specific surface area and pore volume) due to the partial hydrolysis of the MCM-41 silica walls.

### 3.4. Toluene Adsorption from Aqueous Solution

The adsorption kinetics of toluene from aqueous solution by MCM-41 and SBA-15 silicas, before and after the hydrothermal treatments, are reported as supporting information (Figure S11). The kinetics indicates, on average, 4 h as a contact time suitable to reach the adsorption equilibrium. Therefore, all the adsorption isotherms were performed with a sorbent-solute contact time of 4 h.

**Figure 6:**
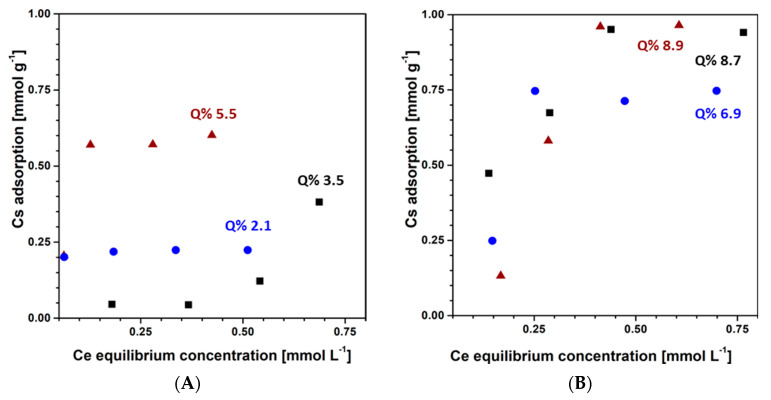
Adsorption isotherms of toluene from aqueous solutions (20–80 mg L^−1^) by MCM-41 (**A**) and SBA-15 (**B**) mesoporous silicas (silica/solution ratio = 10 mg/100 mL at RT) before (black squares) and after hydrothermal treatments (8 h: Red triangles; 36 h: Blue circles). Standard error ≤ 5%. Q% = mg of toluene adsorbed by 100 mg of silica sample (Equation (1)) is reported for the highest adsorption data.

The adsorption isotherms of toluene from aqueous solutions by untreated and treated MCM-41 and SBA-15 samples are reported in Figure 6 Frame A and B, respectively.

As far as the adsorption by the two untreated silicas is concerned, relevant differences were observed both in the isotherm shape and in the adsorption capacity. As a general consideration, MCM-41 retained a minor amount of toluene than SBA-15 within the entire range of toluene initial concentrations (20–80 mg L^−1^). Nevertheless, the affinity of MCM-41 increased at increasing toluene concentrations, showing a type III isotherm. The extension of the adsorption isotherm at higher concentrations was difficult to perform owing to the enhanced toluene volatility from more concentrated aqueous solutions. In contrast, the SBA-15 sample showed an isotherm trend resembling a type I and reached a plateau at 0.94 mmol g^−1^ (Q% 8.7).

On treated MCM-41 samples, toluene adsorption showed a type I isotherm. The isotherm reached a higher plateau on MCM-41_8h (Q% 5.5) than on MCM-41_36h (Q% 2.1, Figure 6 Frame A). Similarly, on treated SBA-15 samples, toluene adsorption reached a higher plateau on SBA-15_8h (Q% 8.9) than on SBA-15_36h (Q% 6.9, Figure 6 Frame B).

These adsorption features can be explained by the structural and chemical changes of the silicas exposed to hydrothermal treatments. MCM-41 tends to reduce its SSA and porosity from 1103 m^2^ g^−1^ and 1.31 cm^3^ g^−1^, respectively, to 888 m^2^ g^−1^ and 1.07 cm^3^ g^−1^ after 8 h of treatment and to 607 m^2^ g^−1^ and 0.87 cm^3^ g^−1^ after 36 h. At the same time, the total number of OH groups increased from 2206 to 7726 after 8 h treatment and then decreased again to 2671 10^18^ g^−1^ after 36 h. After 8 h of treatment, although the reduction in porosity and surface area was observed, the increase in OH groups likely allowed the sorbent to enhance its wettability and resulted in a higher retention of toluene molecules through H-bonding with respect to the pristine silica [8]. Finally, the decrease in SSA and pore volume, together with the reduction in hydroxyl groups, in the sample treated for 36 h, reduced the adsorption of toluene with respect to MCM-41_8h.

The same structural factors can be considered to explain the adsorption feature of the more ordered SBA-15. The silica lost the SSA passing from 761 (untreated) to 689 (SBA-15_8h) and to 634 m^2^ g^−1^ (SBA-15_36h), but, in this material, the hydrothermal treatments increased the pore volume from 0.96 to 1.14 and 1.30 cm^3^ g^−1^, respectively. In addition, the total number of OH groups increased from 2113 to 4065 (SBA-15_8h) and then decreased to 3297 10^18^ g^−1^ (SBA-15_36h). The comparable adsorption of toluene from water of SBA-15 and SBA-15_8h could be explained by a compensation between the SSA decreasing and the increasing of both porosity and OH groups.

These features allow us to conclude that the more ordered SBA-15 shows better performance in adsorbing toluene from the water solution than MCM-41 owing to its structural characteristics and water stability. The water effect on both MCM-41 and SBA-15 porosity and surface groups tends to reduce toluene adsorption from water at prolonged soaking times. Nevertheless, the loss of adsorption capacity for toluene is less critical for SBA-15 than for MCM-41. Thus, SBA-15 silica appears the most promising sorbent for toluene in water media.

From a practical point of view, the typical hydrocarbon concentrations of refinery groundwater can be considered of the order of about 5 mg L^−1^, on average [6]. At these concentrations, SBA-15 results as more effective than MCM-41 in retaining hydrocarbons as it can be clearly observed in Figure 6. Considering the long soaking time of sorbent materials in PRBs (i.e., 12 months as reported by Vignola et al. [37]), SBA-15 seems more favorable than MCM-41 to maintaining its adsorption properties after prolonged water contact time as indicated by the good adsorption performances shown by hydrothermally treated samples (i.e., SBA-15_8h and SBA-15_36h).

## 4. Conclusions

In this work, mesoporous silica with different textural properties (fumed, MCM-41, and SBA-15 silicas) were studied as potential sorbents for toluene from the gas phase, for VOC removal, and from aqueous solution, for groundwater decontamination.

The physico-chemical features of mesoporous silicas were investigated by means of different experimental approaches (i.e., N_2_ adsorption, TGA, FT-IR spectroscopy). The amount of silanols and the textural properties of the samples were analyzed in detail to find a correlation with the toluene adsorption process. 

The most promising materials for toluene removal are found to be ordered mesoporous materials, in which mesopores with dimensions in the 30–90 Å range seem to be important for the adsorption of a significant amount of toluene.

At low pressures (0–5 mbar), the surface silanols play a key role in driving toluene adsorption capacity, due to the O-H·π interactions between silanol species and toluene molecules. Indeed, in this region, SBA-15 silica adsorbs the higher amount of toluene, due to the higher amount of isolated silanol species compared to MCM-41. At low pressure (ca. 27 mbar), MCM-41 has a higher adsorption capacity with respect to the SBA-15 material (ca. +16 Q%). This is because, at this pressure, toluene is adsorbed in the mesopores fraction in the 20–65 Å range, where MCM-41 has a higher mesoporous volume fraction (+63% respect to SBA-15). Contrarily, at higher pressures (>27 mbar), SBA-15 presents a greater adsorption capacity than MCM-41 (up to ca. +12 Q%). Indeed, at this higher pressure, the toluene adsorption is associated with the filling of the second family of homogenous pores in the range between 65 and 100 Å, where SBA-15 has a higher correlated mesoporous volume fraction (+78% compared to MCM-41).

The hydrothermal stability of the mesoporous ordered samples was also investigated, taking into consideration the real conditions that the adsorbent experiences in the hydrocarbon removal process. It was found that the SBA-15 material is less sensitive to the hydrothermal treatment (8 and 36 h in water at 50 °C) compared to MCM-41 due to the higher wall thickness. 

Toluene adsorption from the water solution was negatively affected by the severity of modifications to the structural properties and functional groups of the two mesoporous silicas. Compared to MCM-41, SBA-15 resulted in the most performing material when used for prolonged periods of soaking, owing to its higher wall thickness. Giorgio Gatti Giovanni Golemme

## Figures and Tables

**Table 1:** Main textural features of silica supports used in this study. For MCM-41 and SBA-15, data collected before and after hydrothermal treatment for 8 and 36 h are also reported.

Sample	SSA_BET_^1^ (m^2^ g^−1^)	V_P_^2^ (cm^3^ g^−1^)	V_mesop_^3^ (cm^3^ g^−1^)		t ^4^ (nm)
			20–100 Å		
Fumed Silica	412	1.47	0.31		-
MCM-41	1103	1.31	0.98		0.5
MCM-41_8h	888	1.07	0.79		0.5
MCM-41_36h	607	0.87	0.63		0.5
			20–65 Å	65–120 Å	
SBA-15	761	0.96	0.31	0.55	2.8
SBA-15_8h	689	1.14	0.24	0.68	2.9
SBA-15_36h	634	1.30	0.14	0.88	1.0

^1^ Brunauer-Emmet-Teller (BET) specific surface area (SSA); ^2^ Total pore volume by NLDFT method; ^3^ Volume of mesopores by NLDFT method; ^4^ Wall thickness calculated by using Equations (S2) and (S3) reported in Supporting Information.

**Table 2:** Comparison between the toluene uptake (Q%) at 27 and 45 mbar and the mesoporous volume of MCM-41 and SBA-15 samples.

Sample	Q _at 27 mbar_ (%)	Q _at 45 mbar_ (%)	V_mesop_ (cm^3^ g^−1^)	
			20–65 Å	65–100 Å
Fumed silica	18	71	0.19	0.12
MCM-41	60	78	0.83	0.12
SBA-15	43	90	0.31	0.55

## Supplementary Materials

The following are available online at https://www.mdpi.com/1996-1944/13/12/2690/s1. Figure S1: TEM images of MCM-41 (A) and SBA-15 (B) samples, Figure S2: FT-IR spectra of fumed (curve a), MCM-41 (curve b), and SBA-15 silica (curve c), Figure S3: TGA analysis of fumed (curve a), MCM-41 (curve b), and SBA-15 (curve c) silicas, Xray patterns of samples MCM41 (a), MCM41_8h (a’), MCM41_36h (a’’) (Frame A); SBA15 (b), SBA15_8h (b’), SBA15_36h (b’’) (Frame B), Figure S4: X-ray patterns of samples MCM41 (a), MCM41_8h (a’), MCM41_36h (a’’) (Frame A); SBA15 (b), SBA15_8h (b’), SBA15_36h (b’’) (Frame B), Figure S5: TEM images of MCM41 silica (A) and MCM41_36h (B), Figure S6: N_2_ adsorption (full symbols) and desorption (empty symbols) isotherms of MCM-41 (a), MCM-41_8h (a’), MCM 41_36h (a’’) (A); SBA-15 (b), SBA-15_8h (b’), SBA-15_36h (b’’) (B), Figure S7: FT-IR spectra of MCM-41 (a), MCM-41_8h (a’), MCM-41_36h (a’’) (Frame A); SBA-15 (b), SBA-15_8h (b’), SBA-15_36h (b’’) (Frame B), Figure S8: TGA of MCM-41 (a), MCM-41_8h (a’), MCM-41_36h (a’’) (Frame A); SBA-15 (b), SBA-15_8h (b’), SBA-15_36h (b’’) (Frame B), Figure S9: FTIR spectra of toluene adsorbed at RT on SBA15 sample. Spectrum a was recorded after outgassing the sample for 1 h at 150 °C (before toluene adsorption); spectrum h was recorded after the admission of 30 mbar of toluene; spectra g to b were collected upon decreasing the dosage of toluene, Figure S10: Dependence of toluene uptake to silanols for fumed (blue triangles), MCM-41 (red circles), and SBA-15 silicas (black squares), Figure S11: Adsorption kinetics of toluene from aqueous solution by MCM-41 and SBA-15 mesoporous silicas before and after hydrothermal treatments, Table S1: Amount of SiOH species of MCM-41 and SBA-15 samples before and after hydrothermal treatment for 8 and 36 h at 50 °C, Table S2: IR bands formed after the adsorption of toluene on SBA-15.
