# Supplementary material for: Toluene Adsorption by Mesoporous Silicas with Different Textural Properties: A Model Study for VOCs Retention and Water Remediation
Source: Materials (Basel). 2020 Jun 12;13(12):2690. doi: 10.3390/ma13122690 (PMC7344446; doi:10.3390/ma13122690)
Supplement: Supplementary file 1 [file materials-13-02690-s001.pdf]

materials

MDPI

Supporting Information

Toluene Adsorption by Mesoporous Silicas with Different Textural Properties: A Model Study for VOCs Retention and Water Remediation

Chiara Vittoni [1], Giorgio Gatti [1], Ilaria Braschi [1,2,*], Enrico Buscaroli [2], Giovanni Golemme [3], Leonardo Marchese [1] and Chiara Bisio [1,4,*]

1 Department of Sciences and Technological Innovation and Interdisciplinary NanoSiSTeMI Centre, University of Eastern Piedmont A. Avogadro, viale T. Michel 11, 15121 Alessandria, Italy; chiara.vittoni@uniupo.it (C.V.), giorgio.gatti@uniupo.it (G.G.), leonardo.marchese@uniupo.it (L.M.)

2 Department of Agricultural and Food Sciences, University of Bologna, Viale G. Fanin 44, 40127 Bologna, Italy; enrico.buscaroli2@unibo.it

3 Department of Environmental Engineering, University of Calabria, Via P. Bucci 45A, 87036 Rende, Italy; giovanni.golemme@unical.it

4 CNRSCITEC Istituto di Scienze e Tecnologie Chimiche "Giulio Natta", via G. Venezian 21, 20133 Milano; Italy;

* Correspondence: ilaria.braschi@unibo.it; Tel. +39 051 2096208 (I.B.); chiara.bisio@uniupo.it; Tel.: +39 0131 360216 (C.B.)

Received: 15 May 2020; Accepted: 9 June 2020; Published: date

The morphology and particle size of SBA15 and MCM41 silica samples were investigated by transmission electron microscopy (TEM). The obtained images are reported in Figure S1.

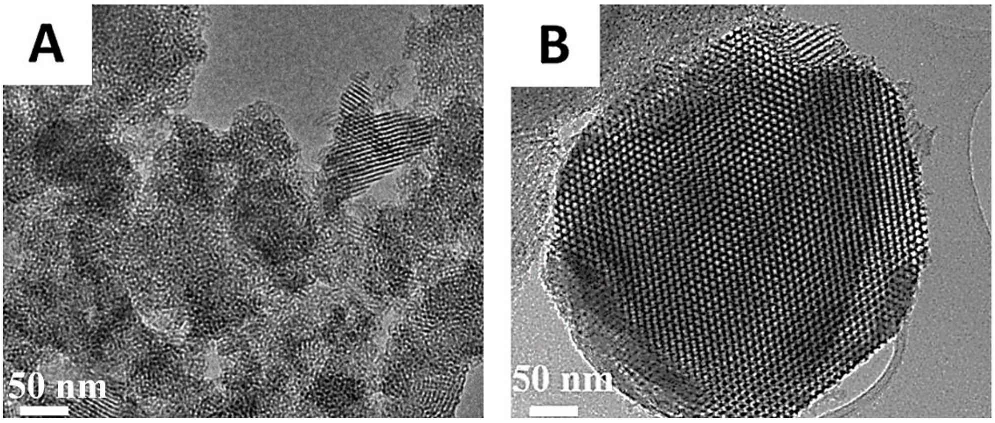

Figure S1. TEM images of MCM41 (**A**) and SBA15 (**B**) samples.

TEM analysis (Figure S1) showed that both MCM41 (A) and SBA15 (B) materials present ordered porosity with hexagonal symmetry. The SBA15 sample is characterized by roundshaped particles with dimensions of about 400 nm, while the MCM41 sample is constituted by particles with dimensions below 50 nm with the presence of a wormhole pore network. The same network for MCM41 has been also observed by Park et al., 2002. Here, they found that the presence of a wormholetype pore system in the MCM41 sample, as well as of a lower condensation degree of the silica walls, leads to the generation of a higher population of structural defects [1]. Surface properties of silica samples were studied by FTIR spectroscopy (Figure S2).

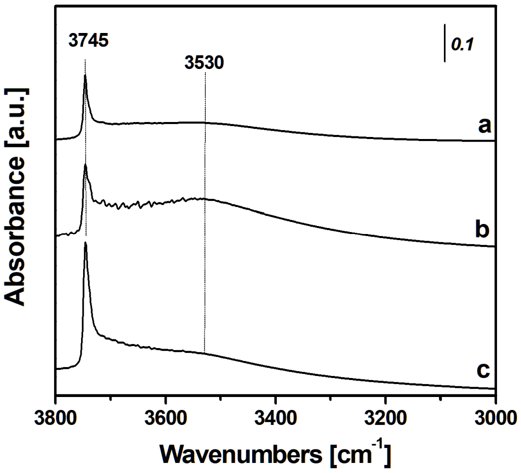

Figure S2. FTIR spectra of fumed (curve a), MCM41 (curve b), and SBA15 silica (curve c).

The spectra of fumed, MCM41, and SBA15 silica samples (Figure S2 curves a, b, and c, respectively) present a sharp peak at ca. 3745 cm^{1} that can be assigned to isolated silanols, and a broad absorption in the 3720–3200 cm^{1} region, with a maximum at around 3530 cm^{1}, that corresponds to hydrogenbonded silanols [2,3]. In the SBA15 sample (Figure S2 curve c), the peak at 3745 cm^{1} appears more intense than those observed in fumed and MCM41 silica samples (curves a and b, respectively), thus suggesting the presence of a higher amount of isolated SiOH species on SBA15.

It is also essential to perform a quantification of the silanols present in the different samples, as this parameter can strongly influence the adsorption of toluene. The quantification was performed by using thermogravimetric analysis (Figure S3).

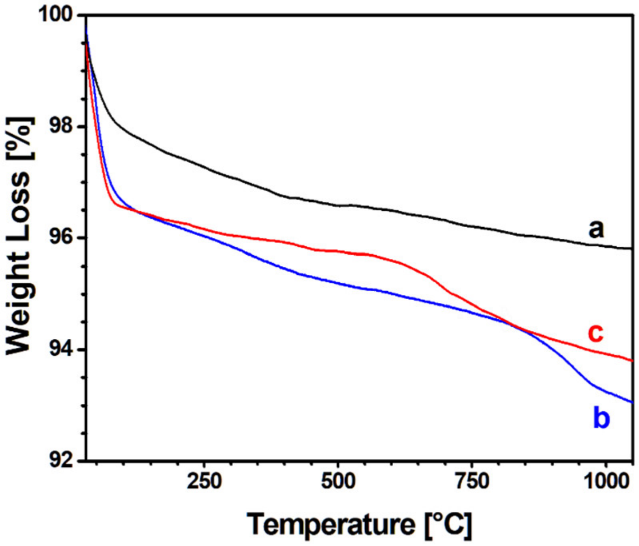

Figure S3. TGA analysis performed at a heating rate of 2°C/min under oxygen flow (100 mL/min) of fumed (curve a), MCM41 (curve b), and SBA15 (curve c) silicas.

The amount of silanols was determined by considering the weight loss within the 150–1050 °C range, which is approximately 1.5, 3.1, and 3.1%, corresponding to 2.5, 2.0, and 2.8 OH/nm^{2} for fumed, MCM41, and SBA15 silica, respectively. The equation used to determine the amount of surface silanols is reported below.

$$\frac{\mathrm{SiOH}}{\mathrm{nm}^2} = \frac{\left(\frac{\%\mathrm{H_2O}}{100 - \%\mathrm{H_2O}} \times \frac{2 \times \mathrm{N_A}}{\mathrm{M.W._{H2O}}}\right)}{\mathrm{S.S.A.} \times 10^{18}} \quad \text{(S1)}$$

Equation S1. Silanol number determination from TGA analysis.

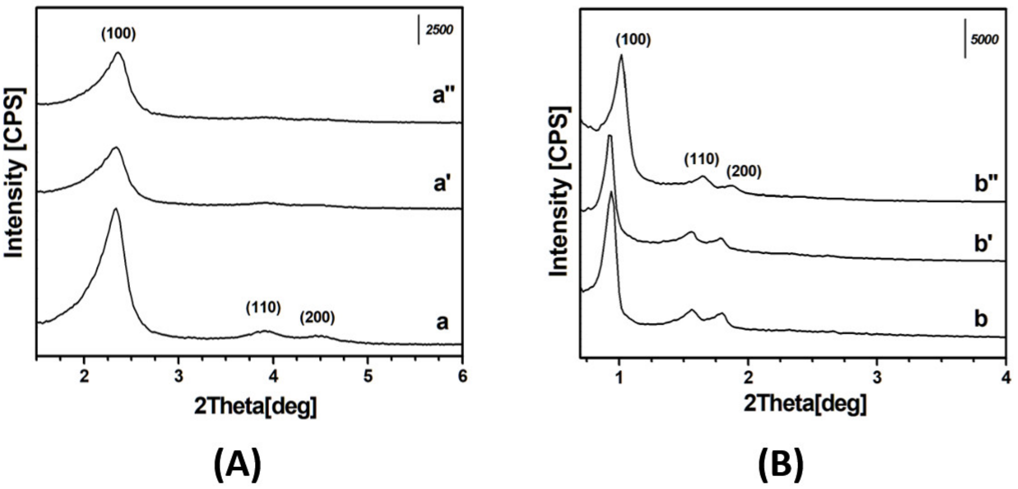

Figure S4. Xray patterns of samples MCM41 (a), MCM41_8h (a'), MCM41_36h (a'') (Frame **A**); SBA15 (b), SBA15_8h (b'), SBA15_36h (b'') (Frame **B**).

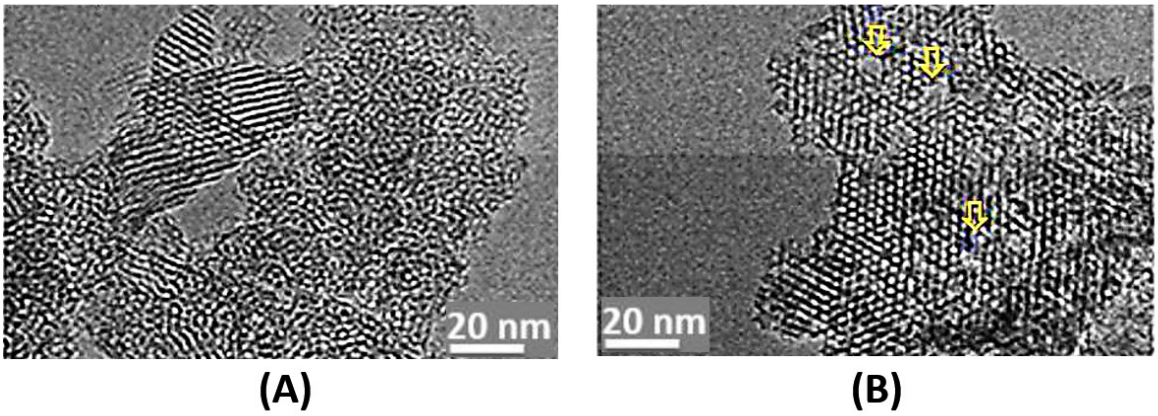

Figure S5. TEM images of MCM41 silica (**A**) and MCM41_36h (**B**).

$$t = a_0 - D \tag{S2}$$

Equation S2. Determination of wall thickness (t) of mesoporous ordered silicas (where D is the mean pore diameter, obtained from N_2 physisorption analysis), while a_0 is the hexagonal unit cell parameter, determined according to the following equation (Equation S.3):

$$a_0 = \frac{2d_{100}}{\sqrt{3}} \tag{S3}$$

Equation S3. Determination of hexagonal unit cell parameter (where d_{100} is the spacing obtained from small-angle XRD analysis by using Bragg's law).

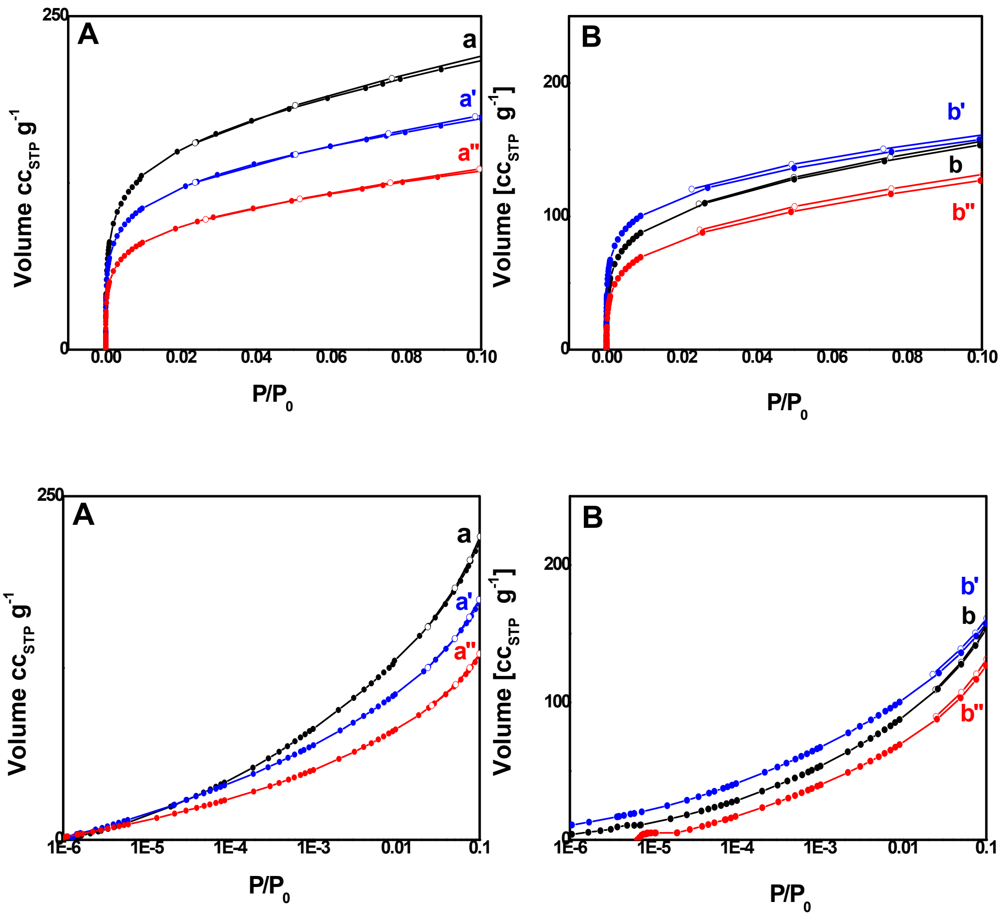

Figure S6. N_2 adsorption (full symbols) and desorption (empty symbols) isotherms of MCM41 (a), MCM41_8h (a′), MCM 41_36h (a′′) (**A**); SBA15 (b), SBA15_8h (b′), SBA15_36h (b′′) (**B**).

Figure S6 shows the initial part of the N_2 sorption isotherms of the porous silicas. The hydrothermal treatment steadily reduces the microporosity of MCM41 (Figure S6A). Figure S6B indicates that the hydrothermal treatment increases the volume of the micropores of SBA15 after 8 h, but then reduces it at longer times (36 h). This observation, together with the reduction in the thickness of the pore walls (Table 1), suggests a densification of the silica walls of SBA15 at the expense of the micropores.

The modification of the silica surface as a consequence of the hydrothermal treatment was also followed by FTIR spectroscopy (Figure S7).

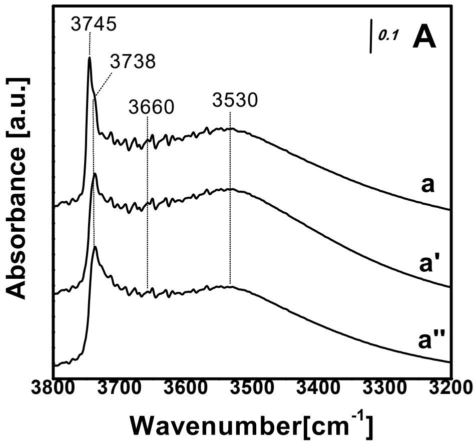

Figure S7. FTIR spectra of MCM41 (a), MCM41_8h (a′), MCM41_36h (a′′) (**A**); SBA15 (b), SBA15_8h (b′), SBA15_36h (b′′) (**B**).

Both MCM41 and SBA15 samples after 8 and 36 h of hydrothermal treatments show a modification of bands related to surface SiOH sites: A decrease in the intensity of the band due to isolated silanols (3745 cm^{1}) is noticed together with a modification of the large band due to Hbonded species. This evolution is another verification of the partial hydrolysis of weak SiOSi siloxanes and the formation of defect sites after hydrothermal treatment [3,4].

The modification of the amount of SiOH species after hydrothermal treatments was determined by thermogravimetric analysis (Figure S8 and Table S1).

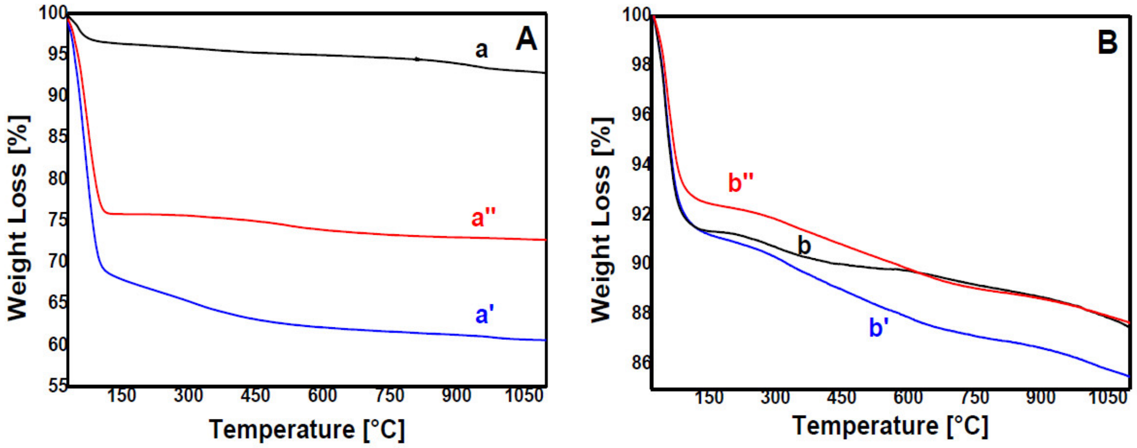

Figure S8. MCM41 (a), MCM41_8h (a′), MCM41_36h (a′′) (Frame **A**); SBA15 (b), SBA15_8h (b′), SBA15_36h (b′′) (Frame **B**).

Table S1. Amount of SiOH species of MCM41 and SBA15 samples before and after hydrothermal treatment for 8 and 36 h at 50 °C.

Sample	N° OH/nm^2		
	**AsPrepared Samples**	**8 h Hydrothermal Treatment**	**36 h Hydrothermal Treatment**
MCM41	2.0	8.7	4.4
SBA15	2.8	5.9	5.2

The increase in the amount of OH groups per squared nm after 8 h of hydrothermal treatment for both materials is an indication of the progressive hydrolysis reactions on the silica surface. After 8 h of treatment, silanol species of the SBA15 sample increase from 2.8 to 5.9 OH/nm^2; prolonging the duration of treatment to 36 h, the silanol species remain around 5.2 OH/nm^2.

The amount of silanols species of MCM41 after 8 h of hydrothermal treatment has a greater increase (from 2.0 to 8.7 OH/nm^2) compared to the SBA15 sample. This is probably due to the fact that MCM41 silica has thinner pore walls than SBA15, and, consequently, more siloxane bonds are sensitive to hydrolysis, leading to longer times to the partial collapse of the structure (Figure S5(B)).

After 36 h of hydrothermal treatment, half of the silanols in MCM41 8h condenses, leaving 4.4 OH/nm^2; the progressive fragmentation into non-porous silica is compatible with the steady reduction of SSA.

FTIR spectra obtained after the adsorption of 30 mbar of toluene and subsequent gradual decrease in toluene pressure on SBA15 silica are reported in Figure S9.

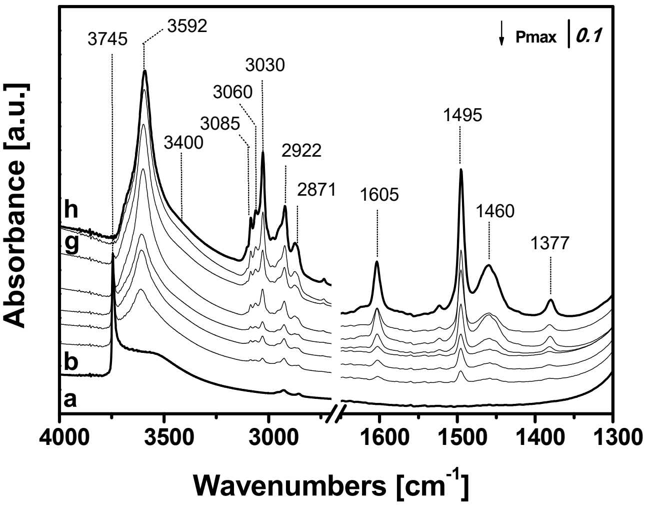

Figure S9. FTIR spectra of toluene adsorbed at RT on SBA15 sample. Spectrum a was recorded after outgassing the sample for 1 h at 150 °C (before toluene adsorption); spectrum h was recorded after the admission of 30 mbar of toluene; spectra g to b were collected upon decreasing the dosage of toluene.

The IR adsorption experiments were carried out starting from high toluene pressure (30 mbar, Figure S9, h) and progressively decreasing (Figure S9, curves gb); however, for the sake of clarity, in the following text, FTIR spectra are described starting from low to high toluene pressure.

Adsorption of small toluene doses, from 0.1 to 9 mbar, on SBA15 silica (Figure S9, curves bg) led to the partial disappearance of the band related to isolated SiOH species at 3745 cm^{-1} and to the concurrent formation of a band at ca. 3592 cm^{-1}. This behavior has already been observed in the case of toluene adsorption on dealuminated zeolites [5]: The band at 3592 cm^{-1} is due to the interaction of isolated silanols present on the silica surface with the aromatic ring of toluene (i.e., O–H·π interactions), the associated $\Delta\upsilon_{OH}$ being ca. 153 cm^{1}. This shift is similar to that observed in zeolites (i.e., 150 cm^{-1}), thus indicating that the strength of the interaction between the silica and toluene is comparable to that between zeolites and toluene [5]. The IR spectra of SBA15 upon contact with toluene also show a broad band with a maximum at ca. 3400 cm^{1}, more evident at pressures higher than 9 mbar. This band is probable due to the interactions of toluene with the hydrogenbonded silanol species present on the surface of SBA15 silica.

Upon the adsorption of toluene, several absorption bands are formed in 31002850 and 16501350 cm^{1} ranges. The attributions of these bands are described in Table S2.

Table S2. IR bands formed after the adsorption of toluene on SBA15 [5].

Frequency (cm^{-1})	Vibration Mode
3085, 3060 and 3030	ν CH aromatic ring
2922 and 2871	ν_s and ν_{as} CH toluene methyl groups
1605	quadrant stretching mode of monosubstituted ring C=C bond

1495	semicircular stretching vibration monosubstituted aromatic ring
1460 and 1377	outofphase and inphase deformations of methyl group

Increasing the toluene amount (until 30 mbar, Figure S9, curve h), it is also possible to observe a slight increase in the intensity of the bands related to the toluene molecules. This indicates that the adsorption is likely driven by van der Waals interactions between the silica walls and toluene molecules (hostguest interactions) and among toluene molecules (guestguest interactions).

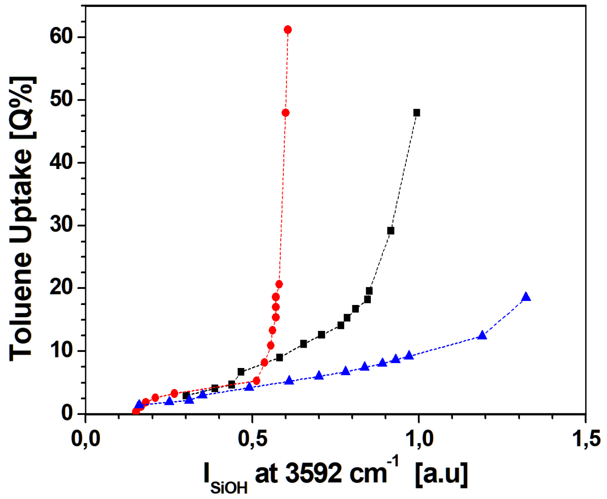

Figure S10. Dependence of toluene uptake to silanols for fumed (blue triangles), MCM41 (red circles), and SBA15 silicas (black squares).

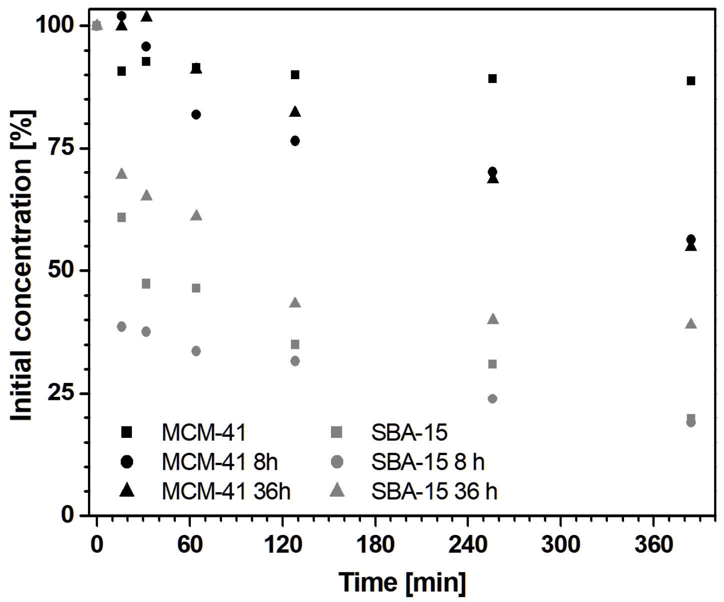

Figure S11. Adsorption kinetics of toluene from aqueous solution (80 mg L1) by MCM41 and SBA15 mesoporous silicas before and after hydrothermal treatments at the silica/solution ratio of 10 mg/100 mL. Standard error ≤ 5%.

References

1. Park, D.-W.; Choi, S.-D.; Choi, S.-J.; Lee, C.-Y.; Kim, G.-J. Asymmetric Epoxidation of Styrene on the Heterogenized Chiral Salen Complexes Prepared from Organo-Functionalized Mesoporous Materials. *Catal. Lett.* **2002**, *78*, 145–151, doi:10.1023/a:1014945926583.
2. Burneau, A.; Barres, O.; Gallas, J.P.; LaValley, J.C. Comparative study of the surface hydroxyl groups of fumed and precipitated silicas. 2. Characterization by infrared spectroscopy of the interactions with water. *Langmuir* **1990**, *6*, 1364–1372, doi:10.1021/la00098a008.
3. Gallas, J.-P.; Goupil, J.-M.; Vimont, A.; LaValley, J.-C.; Gil, B.; Gilson, J.-P.; Miserque, O. Quantification of Water and Silanol Species on Various Silicas by Coupling IR Spectroscopy and in-Situ Thermogravimetry. *Langmuir* **2009**, *25*, 5825–5834, doi:10.1021/la802688w.
4. Hahn, M.W.; Copeland, J.R.; Pelt, A.H.V.; Sievers, C. Stability of Amorphous Silica-Alumina in Hot Liquid Water. *ChemSusChem* **2013**, *6*, 2304–2315, doi:10.1002/cssc.201300532.
5. Sacchetto, V.; Bisio, C.; Olivera, D.F.O.; Paul, G.; Gatti, G.; Braschi, I.; Berlier, G.; Cossi, M.; Marchese, L. Interactions of Toluene and n-Hexane on High Silica Zeolites: An Experimental and Computational Model Study. *J. Phys. Chem. C* **2015**, *119*, 24875–24886, doi:10.1021/acs.jpcc.5b08380.
